# Differentiation Analysis on Carbon Emission Efficiency and Its Factors at Different Industrialization Stages: Evidence from Mainland China

**DOI:** 10.3390/ijerph192416650

**Published:** 2022-12-11

**Authors:** Lijie Wei, Zhibao Wang

**Affiliations:** College of Geography and Environment, Shandong Normal University, Jinan 250358, China

**Keywords:** carbon emission efficiency (*CEE*), industrialization, super-SBM model, mainland China

## Abstract

Industrial production is currently the main source of global carbon emissions. There are obvious differences in regional carbon emission efficiencies (*CEE*) at different industrial stages. We investigate *CEE* and explore its factors in mainland China at different industrialization stages from 2008-2020 using the super-SBM model with an undesirable output and the STIRPAT model. There is significant spatial heterogeneity in regional *CEE*, with gaps gradually widening. *CEE*’s spatial heterogeneity in mid-industrialized provinces is narrowing, while in late-industrialized and post-industrialized provinces, it is widening. *CEE*’s factors also differ in provinces at different industrialization stages. At the mid-industrialization stage, the industrial structure (*IS*) is the dominant factor, while population urbanization (*PU*) is dominant at the late-industrialization stage, and both *PU* and *IS* are dominant at the post-industrialization stage. Based on *CEE*’s characteristics at different industrialization stages, we propose suggestions for green development.

## 1. Introduction

Carbon emissions have become a major constraint to regional development, limiting regions whose increases are necessitated by COVID-19 epidemic normalization, economic recovery, and enormous energy demands. Global CO_2_ emissions exceeded 33.8 billion tons in 2021, demonstrating a growth rate of 5.00%. As the largest global carbon emitter, China’s carbon emissions reached 10.5 billion tons in 2021. In terms of global CO_2_ emissions in 2021 (Global CO_2_ emission in 2021: Asia Pacific accounts for more than half of carbon emissions, China tops the world in carbon emissions: https://www.sohu.com/a/578797859_120950203 (accessed on 11 October 2022)). As a developing country, China still relies on extensive energy consumption. As such, China’s carbon emissions are still continuously increasing. For sustainable development, China strives to reach “carbon peaking” by 2030 and “carbon neutrality” by 2060. At the 20th National Congress of the Communist Party of China, it was proposed that China should actively and steadily promote “carbon peaking” and “carbon neutrality” and actively respond to the global governance of climate change.

Carbon emission efficiency (*CEE*) measures regional contributions (e.g., to climate change) and reflects the ratio of the production relationship between the minimum CO_2_ emissions created and the maximum economic output [1]. In terms of research scales, countries [2,3,4], regions [5,6,7], and some particular units [8] are some of the main objects of previous studies, while different industries (e.g., industrial structure, transportation industry, and environmental regulation) are among the main directions [9,10]. However, due to differentiated resource endowments, interregional economies are uneven. As a result, regional carbon emissions may also differ, yet there are fewer studies [11] on cross-sectional comparisons [12] of the same geographical units, which would help to provide tailored suggestions for regional characteristics. A single index measurement can reflect the local *CEE* situation [13,14,15,16] but often ignores the relationships and influences of other indices, such as the region, capital, energy, and population’s influences on its *CEE* [17]. Various input and output aspects have become the main methods of research to measure CEE more scientifically and comprehensively. Envelope analysis [18], super-efficient DEA [19], the stochastic frontier method [20], non-radial directional distance function [21], and the SBM [22] method of window analysis have become more frequently applied. Meanwhile, spatial autocorrelation analysis [23], kernel-density estimation [24], the Gini coefficient [25], the coefficient of variation [26], and the Thiel index [27] are often used to investigate *CEE*’s spatial heterogeneity, correlation, and spatial spillover. Additionally, structural-decomposition analysis (SDA) [26], exponential decomposition analysis [28], and the logarithmic mean Divisia index (LMDI) [29,30] are used to explore *CEE*’s factors. Due to the differences in research units or perspectives, even similar indicators may show different results.

New research findings on carbon emissions and *CEEs* are emerging, and their research perspectives, contents, and methods are more systematic and comprehensive. They are needed to explore the situations and factors of regional carbon emissions and *CEEs* of different types, categories, and characteristics. As such, our paper will have two main contributions: (1) The super-SBM model with undesirable output measures of *CEEs* at different industrialization stages, which will provide a valid and typical reference for other global regions; (2) Based on the STIRPAT model, we explore *CEE*’s dominant factors at different industrialization stages, which extends the research paradigm of *CEE*’s differential analysis and promotes sustainable development and other related studies.

## 2. Data and Methodology

### 2.1. Data Sources and Pre-Processing

We selected 30 provinces in mainland China (Tibet, Hong Kong, Macao, and Taiwan were not included in our study due to data availability). Data on the urban employed labor forces were from the China Population and Employment Statistical Yearbook (2009–2021). Data on the total national energy consumption were from the China Energy Statistical Yearbook (2009–2021) and the Statistical Bulletin of National Economic and Social Development (2009–2021*)*. Data on the capital stocks, GDPs, population capacities, population urbanizations, industrial structures, and foreign investments were from the China Statistical Yearbook (2009–2021) and local statistical bulletins, and the number of patents granted was from the State Intellectual Property Office (https://www.cnipa.gov.cn/ (accessed on 5 September 2022)). Carbon emissions data from 2008 to 2019 were calculated with reference to the IPCC Guidelines for National Greenhouse Gas Emissions Inventories, 2006 Edition (IPCC 2006). Carbon emissions in 2020 were projected by a gray model with reference to previous years’ data.

#### 2.1.1. Selection of Variables

By combining previous studies [31,32,33,34,35], technology, economic development, demographic transition, and resource dependence were shown to affect *CEE*. As such, *CEE* was selected as the explained variable, while economic development (*ED*), industrial structure (*IS*), population capacity (*PC*), population urbanization (*PU*), foreign investment (*FI*), total energy consumption (*EC*), and technology (S*T*) were selected as explanatory variables (Table 1).

We completed a panel regression analysis with Stata16. To eliminate the possible influence of heteroskedasticity on upper models, we took the logarithms of all variables. The factors’ regression analysis was conducted using the random effects model (RE) and fixed effects model (FE). The Hausman test showed that the fixed effects model (FE) was more appropriate for our research of nationwide and mid-industrialization and late-industrialization stages. A high goodness-of-fit coefficient formed in the fixed effects model (FE), two-way fixed-effects model (FE-tw), and two-way fixed model (FE-tw). Thus, a two-way fixed model (FE-tw) was selected. At the post-industrialization stage, the random effects model (RE) was more appropriate for our research. To prevent “pseudo-regression”, the panel series were tested for stationarity using an HT test. The results (Table 2) showed that all panel data passed the significance test.

#### 2.1.2. Industrialization Stages Division Criteria

We measured the local industrialization level using five indicators, namely: economic development, industrial structure, manufacturing structure, spatial structure, and employment structure (Table 3). The evaluation values, numbers, and weights of individual indicators were counted and calculated. Thus, a composite index of local industrialization level was calculated using the additive synthesis method.

The results show that all 30 provinces were at the mid-industrialization stage and beyond in 2020 (Table 4). Hainan, Heilongjiang, Guangxi, and the other three provinces were at the mid-industrialization stage. Hebei, Inner Mongolia, Jilin, and 18 other provinces were at the late-industrialization stage. Fujian, Shandong, Hubei, Guangdong, Chongqing, and Zhejiang ranked at the top at the late-industrialization stage, where industrialization was significantly improved. Beijing, Tianjin, and Shanghai were already at the post-industrialization stage because of their strong economy and long industrialization history.

### 2.2. Research Methods

#### 2.2.1. Super-SBM Model of Undesirable Output

The traditional DEA model [36] measures efficiency from a single perspective and ignores the possible slackness of input and output, which can cause specific measurement errors. The non-radial, non-angle SBM model solves some problems of the traditional DEA model [37]. However, this model usually results in the efficiency value of multiple decision-making units being 1, making it impossible to further distinguish each unit’s efficiency. The super-efficient DEA and SBM models form the super-efficient SBM model [38], which can handle situations where multiple decision units have an efficiency value of 1 and can distinguish the efficiency of effective decision units. We measured *CEE* in China’s provinces using the Super-SBM model with an undesirable output [39] via Matlab2020b. The *CEE* index system consists of three input indicators, namely: capital, labor, and energy. The output indicators include desired and undesirable output factors (Table 5).

#### 2.2.2. Measuring CEE’s Regional Differences

The coefficient of variation (*CV*), Gini coefficient (*G*), and Thiel index (*T*) were chosen to explore *CEE*’s overall differences nationwide and at different industrialization stages. In the Gini coefficient, *CEE* in each province needs to be ranked in descending order and then calculated. The formulas are as follows.

$$CV=\frac{\sqrt{\sum_{i=1}^{n}{\left(Z_{i}-\bar{Z}\right)}^{2}/n}}{\bar{Z}}$$

$$G=\frac{2}{\bar{Z}n^{2}}(Z_{1}+2Z_{2}+3Z_{3}+\cdots +NZ_{n})-\frac{n+1}{n}$$

$$T=\frac{1}{n}\sum_{i=1}^{n}\frac{Z_{i}}{\bar{Z}}\ln\frac{Z_{i}}{\bar{Z}}$$

where *Z_i_* denotes *CEE* in province *I*; $\sqrt{\sum_{i=1}^{n}{\left(Z_{i}-\bar{Z}\right)}^{2}/n}$ is standard deviation, which denotes the square root of the arithmetic mean of *CEE* in province *i* and China’s average; and $\bar{Z}$ denotes the mean of the overall *CEE*.

#### 2.2.3. Econometric Model

IPAT is an equation for studying environmental factors [40]. Based on IPAT, the STIRPAT model was proposed to lift the model’s limitations due to homogeneous linear variation. The STIRPAT model is as follows:

$$I=aP^{b}A^{c}T^{d}e$$

where *I*, *P*, *A*, and *T* denote environmental impact, population, affluence, and technology, respectively; *a* is the model coefficient; *b*, *c*, and *d* are indices to be taken into account; and *e* is the error term.

Heteroskedasticity may have an impact on the model results. Therefore, we transformed the STIRPAT model into a logarithmic form.

lnI=lna+blnP+clnA+dlnT+e

Additionally, factors such as industrial structure (*IS*), foreign investment (*FI*), energy consumption (*EC*), and population urbanization (*PU*) were added to the model on top of the basic model consistently.

$$\ln CEE=\alpha_{it}+\mu_{1}\ln ST_{it}+\mu_{2}\ln ED_{it}+\mu_{3}\ln PC_{it}+\mu_{4}\ln PU_{it}+\mu_{5}\ln IS_{it}+\mu_{6}\ln EC_{it}+\mu_{7}\ln FI_{it}+\varepsilon_{it}$$

where *CEE* denotes carbon emission efficiency; *ST* denotes technology; *ED* denotes GDP, *PC* denotes population capacity; *PU* denotes population urbanization; *IS* denotes industrial structure; *EC* denotes energy consumption; *FI* denotes foreign investment; *α* denotes the intercept term; *ε* is a random error term; and *I* and *t* denote the observation province and observation year, respectively. *μ* is the coefficient of the explanatory variable.

## 3. Results Analysis

### 3.1. CEE’s Spatio-Temporal Evolution

#### 3.1.1. Temporal Evolution

**Figure 1:**
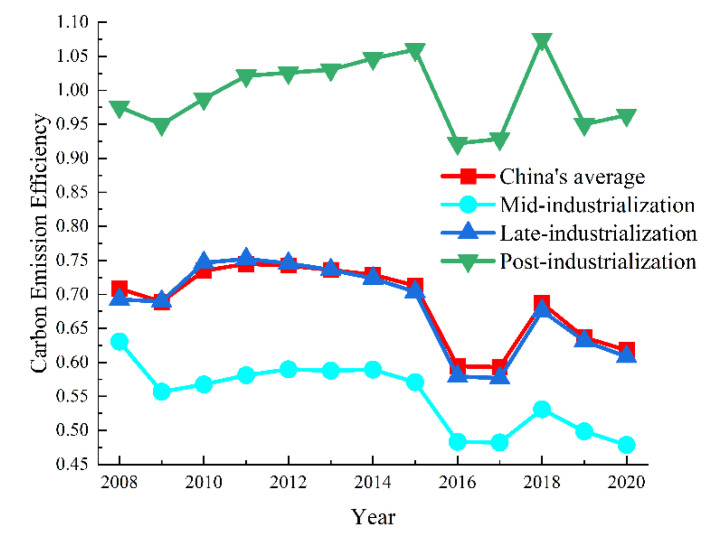
*CEE* at different industrialization stages.

Based on the Super-SBM model with undesirable output, *CEE* was calculated in China’s 30 provinces at various industrialization stages during 2008–2020 (Figure 1). There was a significant difference in *CEE* at various industrialization stages. During 2008–2020, *CEE* showed a fluctuating evolution pattern, namely: *CEE* _post-industrialization_ > *CEE* _late-industrialization_ > *CEE* _mid-industrialization_. *CEE* remained between 0.922 and 1.075 at the post-industrialization stage, significantly higher than China’s average. *CEE* ranged from 0.577 to 0.752 at the late-industrialization stage. China’s highest *CEE* average was 0.745 in 2011, while the lowest was 0.593 in 2017. At the mid-industrialization stage, the highest *CEE* was 0.631 in 2008, while the lowest was 0.479 in 2020. At the late-industrialization stage, *CEE* reached a maximum of 0.752 in 2011 and a minimum of 0.577 in 2017. At the post-industrialization stage, *CEE* reached a maximum of 1.075 in 2018 and a minimum of 0.922 in 2016.

#### 3.1.2. Spatial Evolution

**Figure 2:**
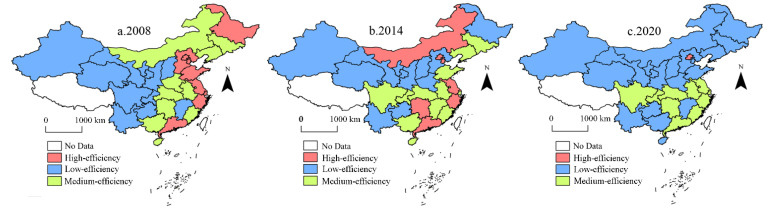
*CEE* in mainland China during 2008–2020.

We classified *CEE* in mainland China’s 30 provinces into high-efficiency, medium-efficiency, and low-efficiency zones via K-means cluster analysis (Figure 2). Overall, China’s *CEE* was in constant change, with significant provincial differences. The high-efficiency zone showed a geographical characteristic of a “concentration-decentralization-contraction” phase. The medium-efficiency zone was mainly concentrated in the central region and gradually clustered to the southeast. The low-efficiency zone spread from the west to the northeast and southwest. In 2008, the high-efficiency zone was mainly concentrated in China’s eastern coastal region. The medium-efficiency zone was scattered. The low-efficiency zone was mainly concentrated in the middle-upper reaches of the Yellow River and the upper reaches of the Yangtze River. In 2014, the high-efficiency zone was gradually dispersed. The medium-efficiency zone was concentrated in the Yangtze River basin and distributed closer to the southeast. The low-efficiency zone extended to the northeast. In 2020, only Beijing was still in the high-efficiency zone. The medium-efficiency zone was concentrated in the Yangtze River basin and closer to the southeast. The low-efficiency zone extended to the northeast, from which Yunnan and Guizhou gradually withdrew.

**Figure 3:**
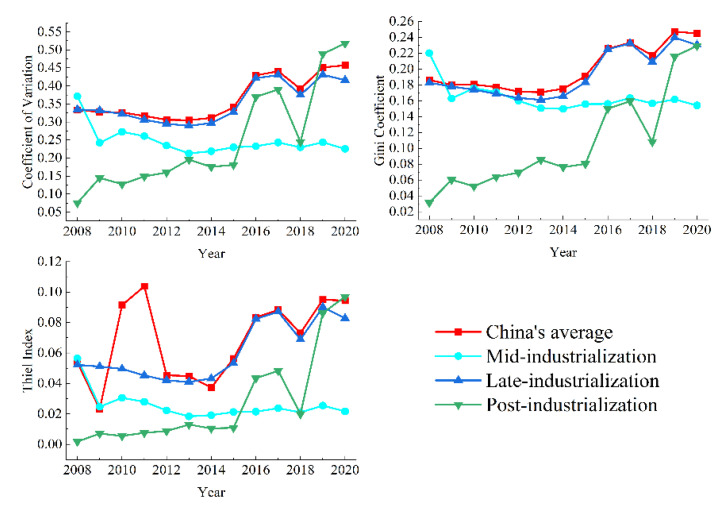
*CEE*’s regional differences at different industrialization stages.

Based on *CEE* data during 2008–2020, the variability of *CEE* by year and by industrialization was analyzed (Figure 3). The variability of *CEE* in China’s provinces generally showed a fluctuating growth trend during 2008–2020. The Thiel index (*T*), Gini coefficient (*G*), and coefficient of variation (*CV*) showed that China’s *CEE* has a folding pattern. *G* ranged from 0.171 to 0.247. The highest value of *T* was 0.104, while the lowest value was 0.023. The highest value of *CV* was 0.457 in 2020, while the lowest value was 0.305 in 2013.

The trends in *CV*, *G*, and *T* were generally consistent at the same industrialization stage. The opposite is true at different industrialization stages. Meanwhile, there were some differences in provincial *CEE* at different industrialization stages (Figure 3). At the mid-industrialization stage, China’s provinces were more homogeneous, and internal differentiation gradually narrowed at the mid-industrialization stage, *T* ∈ [0.018, 0.056]. The variability of provincial *CEE* showed a narrowing and then widening characteristic at the late-industrialization stage, *T* ∈ [0.041, 0.090]. At the post-industrialization stage, *CEE*’s differentiation was smaller, while the regional differentiation increased over time. The highest value of *T* was 0.097 in 2020, while the lowest value was 0.002 in 2008. Although they were at the same industrialization stage, there were large differences in economic and energy aspects.

### 3.2. Factor Analysis

#### 3.2.1. Analysis of the Overall Results

The panel regression results showed that an increase in economic development (*ED*), industrial structure (*IS*), and foreign investment (*FI*) contributed to the improvement in *CEE*. Every 1% increase in economic development (*ED*) increased *CEE* by 0.252%, while every 1% increase in industrial structure (*IS*) increased CEE by 0.712%. A 1% increase in foreign investment (*FI*) contributed to a 0.040% increase in *CEE*. In contrast, population capacity (*PC*) and energy consumption (*EC*) had a significant negative impact on *CEE* and were not conducive to improving *CEE*. At the 1% significance level, every 1% increase in population capacity (*PC*) decreased *CEE* by 0.210%. At the 10% significance level, every 1% increase in energy consumption (*EC*) decreased *CEE* by 0.023% (Table 6).

Currently, China’s economy is rapidly developing. When the economy reaches a certain level, regional development will transfer from pursuing development speed to development quality. In China in 2021, CO_2_ emissions of 10^4^ RMB GDP decreased by 3.8% compared to 2020. From 2017 to 2021, China’s CO_2_ emissions of 10^4^ RMB GDP declined continuously and exceeded the climate action target in 2020, ahead of schedule aiming for 2019. The expansion of population capacity (*PC*) puts pressure on urban transport infrastructure. Traffic congestion and heavy transport consume a lot of energy and increase carbon emissions, which are not conducive to *CEE*. An excessive increase in population capacity (*PC*) can also damage the local environment. Coal is dominant in China’s primary energy consumption. By 2021, the share of non-fossil energy in China’s primary energy consumption reached 16.60%, and China decreased energy consumption per unit of GDP by 26.40% compared to 2012, saving 1.4 billion tons of standard coal. Relying on extensive coal consumption for economic development is not conducive to green and low-carbon development, which reflects the importance and necessity of transforming energy consumption (*EC*).

#### 3.2.2. Analysis of Zoning Results

At the mid-industrialization stage, economic development (*ED*), population urbanization (*PU*), and industrial structure (*IS*) all passed the significance test and had a significant positive relationship with *CEE* (Table 7). At the 1% significance level, every 1% increase in economic development (*ED*) increased *CEE* by 0.146%, every 1% increase in population urbanization (*PU*) increased *CEE* by 0.735%, and every 1% increase in industrial structure (*IS*) increased *CEE* by 0.748%. Population capacity (*PC*) is not conducive to improving *CEE*. Every 1% increase in population capacity (*PC*) decreased *CEE* by 0.163% at the 5% significance test. Among all positive factors, industrial structure (*IS*) is the dominant factor of *CEE* at the mid-industrialization stage.

At the late-industrialization stage, technology (*ST*) passed the 5% significance test and showed a significant negative correlation with *CEE* (Table 7). Every 1% increase in technology (*ST*) decreased *CEE* by 0.107%. Economic development (*ED*), population urbanization (*PU*), and industrial structure (*IS*) all passed the significance test and contributed to improving *CEE*. Every 1% increase in economic development (*ED*) increased *CEE* by 0.263%, while every 1% increase in population urbanization (*PU*) increased *CEE* by 1.245%. At the late-industrialization stage, population urbanization (*PU*) was *CEE*’s main factor.

At the post-industrialization stage, all variables, except foreign investment (*FI*), passed the significance test and showed a significant relationship with *CEE* (Table 7). Among these, technology (*ST*), economic development (*ED*), and industrial structure (*IS*) contributed to the improvement in *CEE*. Every 1% increase in technology (*ST*) increased *CEE* by 0.262%, every 1% increase in economic development (*ED*) increased *CEE* by 0.165%, and every 1% increase in industrial structure (*IS*) increased *CEE* by 1.024%. Population capacity (*PC*), population urbanization (*PU*), and energy consumption (*EC*) hindered the improvement in *CEE*. Every 1% increase in population capacity (*PC*) decreased *CEE* by 0.985%, every 1% increase in population urbanization (*PU*) decreased *CEE* by 3.744%, and every 1% increase in energy consumption (*EC*) decreased *CEE* by 0.189%. Among all significant positive factors, industrial structure (*IS*) was the most dominant, while among all significant negative factors, population urbanization (*PU*) was dominant.

However, at different industrialization stages, there are many similarities regarding *CEE*’s factors. Both economic development (*ED*) and industrial structure (*IS*) are significant. Population capacity (*PC*) has a significant negative impact at the mid- and post-industrialization stages, which significantly differ. Technology (*ST*) has a significant negative impact on *CEE* in the provinces at the late-industrialization stage but has a significant positive effect at the post-industrialization stage. Population urbanization (*PU*) passes the significance test at all different industrialization stages. Population urbanization (*PU*) has a positive impact and enhances *CEE* at the mid- and late-industrialization stages, which is detrimental to *CEE* at the post-industrialization stage.

There is a difference in the impact of technology (*ST*) on *CEE* in provinces at different industrialization stages. At the late-industrialization stage, every 1% increase in technology (*ST*) decreased *CEE* by 0.107% but increased *CEE* by 0.262% at the post-industrialization stage. Economy, industry, and space differ among provinces at the late-industrialization stage. Urban construction and economic development still require a significant amount of capital investment. Technology (*ST*) requires much more capital and human resources to drive progress and improvement. Capital investment far outweighs economic output generated by technology (*ST*), while R&D requires more energy consumption (*EC*), all of which will increase provincial financial pressure to the detriment of local development at the late-industrialization stage. Meanwhile, pursuing economic development (*ED*) requires the intensive use of energy consumption (*EC*). Therefore, an increase in technology (*ST*) requires more energy consumption (*EC*), decreasing *CEE*.

Population urbanization *(PU)* significantly positively affected *CEE* at the mid- and late-industrialization stages. A 1% increase in population urbanization (*PU*) increased *CEE* by 0.735% at the mid-industrialization stage, and increased *CEE* by 1.245% at the late-industrialization stage. At the post-industrialization stage, population urbanization (*PU*) had a significant negative effect on *CEE*. Every 1% increase in population urbanization (*PU*) will decrease *CEE* by 3.744%. As population urbanization (*PU*) rises, human capital sustains local economic development. Meanwhile, human capital drives sustainable economic development, transitioning towards low-carbon development and clean production. It also leads to changes in urban residents’ consumption, shifting towards low-carbon, environmental protection, and cleanliness. The shift in urban residents’ consumption has increased energy use efficiency, reduced carbon emissions, and promoted *CEE*. Further increases in population urbanization (*PU*) will burden urban construction. Urban residents directly or indirectly increase energy consumption (*EC*) and carbon emissions, which is not conducive to improving *CEE*.

#### 3.2.3. Robustness Test

There may be issues such as omitted variables in the model, resulting in endogeneity issues affecting the model’s results and the stability of the regression results. Based on previous research [41], we chose five representative quantile points of 10%, 25%, 50%, 75%, and 90% to complete a panel quantile regression model (Table 8). The results showed that the nature and significance of each variable’s effects were generally consistent with the overall regression results, indicating that our study’s results are reliable and stable.

## 4. Discussion

### 4.1. Problem and Recommendations

(1) At the mid-industrialization stage, population migration leads to a decrease in the population urbanization rate, which is not conducive to *CEE*. At this stage, every 1% increase in population urbanization increases *CEE* by 0.967%, and an increase in urbanization increases *CEE*. The scale of migration across provinces in 2020 was 125 million persons, an increase of 38.96 million persons or 45.37% over 2010. At the mid-industrialization stage, China’s provinces face a population exodus, which is not conducive to *CEE*. Provinces need to attract the population back and bring in high-level talent at the mid-industrialization stage. Human capital can accelerate the upgrading and transformation within local enterprises through the skills and proficiency of the educated population [42]. It can apply advanced technology and production methods to local practices, which can help to increase the efficiency of enterprises’ production and operation, improve the regional input-output situation, and thus improve *CEE* [43].

(2) Enhancement in technology innovation inhibits improvement in *CEE*, which is significantly related to the input and output situation in technical innovation. At the late-industrialization stage, every 1% increase in technology decreases *CEE* by 0.107%. China’s innovation input index was 219.0 in 2021, while its innovation output index was 353.6. Environmental protection and low-carbon development should receive attention in some provinces at the late-industrialization stage, and technical innovation should be improved in some provinces at the late-industrialization stage [44]. Enterprises and research institutes should increase capital investment in R&D and improve the efficiency of transforming technical achievements [12].

(3) At the post-industrialization stage, some provinces advance China in terms of socioeconomic development. At the post-industrialization stage, a 1% increase in population urbanization decreases *CEE* by 3.744%. Population urbanization is not conducive to *CEE*. China is home to a high amount of talent, capital, and immigrants. The population urbanization rate reached 64.72% in China in 2021 and was above 80.00% in Beijing, Tianjin, and Shanghai. Urban scale structure and urban development should be optimized [45]. Environmental awareness and guiding residents to develop green consumption and low-carbon lifestyles should be strengthened [46]. Based on provincial characteristics, China needs to develop high-tech, low-carbon green industries to improve urban functions, reduce carbon emissions, and thus improve local *CEE* [47].

(4) It is necessary to develop targeted efficiency improvement strategies according to local conditions. Local governments should guide enterprises to shift to a low-carbon model in their production and operations [20]. In pursuing low-carbon development, it is essential to provide capital, technical, and human resources assistance to provinces with low *CEE* [48]. Provinces should accelerate regional communication, fully use the technology of energy conservation and emission reduction and accelerate regional industrial upgrading based on local resources [49]. China should strengthen its environmental laws and institutions and enhance the enforcement of environmental policies [50]. Foreign investment can bring advanced production technology experience, enhance international cooperation, and take advantage of the “technology spillover” effect of foreign enterprises in energy conservation and emission reduction to raise the environmental awareness of domestic enterprises [51]. Additionally, air pollution, such as PM_2.5_ [52], is an important external factor for *CEE*. Interregional exchange and learning can improve *CEE* and reduce regional differences.

### 4.2. Research Perspectives

In this paper, we investigated *CEE*’s spatial heterogeneity and its factors at different industrialization stages. The large variability among industrialization stages proves our study is of theoretical and practical value. The *CEE* analysis paradigm based on differentiated industrialization stages is our greatest theoretical innovation and contribution to research. Based on the differentiated characteristics and existing problems, we propose suggestions for human capital, technical innovation, and urbanization construction, which can help promote efficient and green development. However, there are still certain shortcomings or limitations to our study. We used provinces as the basic study unit and only explored industrialization stages. Additionally, provincial *CEE* does not reflect its specific actual situation in a comprehensive and detailed manner. Some of China’s provinces (e.g., Inner Mongolia) span a wide range of regions, with significant disparities in the economy, carbon emissions, energy consumption, population, etc. The data on carbon emissions are relatively old. Furthermore, there are still some missing data from recent years. In this paper, we predict carbon emissions using the model. However, in terms of actual measurements, carbon emissions are affected by other factors. Therefore, the prediction does not reflect the actual local situation effectively. These are the research aspects that we will strive to improve in the future.

## 5. Conclusions

By constructing panel regression models at different industrialization stages, we explore *CEE*’s various factors. China’s *CEE* shows a significant fluctuation, with provincial *CEE* increasing at the post-industrialization stage and decreasing at other industrialization stages. *CEE* is characterized by *CEE* _post-industrialization_ > *CEE* _China’s average_ > *CEE* _late industrialization_ > *CEE* _mid-industrialization_. Provincial *CEE*’s differentiation gradually decreases at the mid-industrialization stage, which keeps increasing at the late industrialization and post-industrialization stages. Among *CEE*’s factors, industrial structure (*IS*), economic development (*ED*), population capacity (*PC*), and population urbanization (*PD*) are the main factors. There are differences in the influence of factors at different industrialization stages. Industrial structure (*IS*) is the most important factor of *CEE*, especially during the mid-industrialization. Population urbanization (*PU*) is the most significant factor, while whose impact is opposite at the late- and post-industrialization stages.

## Figures and Tables

**Table 1:** Selected variables.

Variables	Indicators	Unit	Meaning
Explained variable	Carbon emission efficiency (*CEE*)	-	Measurement of Super-SBM model with undesirable output
Explanatory variables	Technology (*ST*)	PC	Number of patents granted
	Economic development (*ED*)	10^8^ RMB	Gross Domestic Product
	Population capacity (*PC*)	(people/km^2^)	District population/area of jurisdiction
	Population urbanization (*PU*)	%	Urban population/year-end resident population
	Industrial structure (*IS)*	%	Secondary industry added value/GDP
	Energy consumption (*EC*)	10^4^ tons of standard coal	Total energy consumption
	Foreign investment (*FI*)	%	Foreign direct investment/GDP

**Table 2:** Descriptive statistics for variables.

Variables	Mean	Standard Deviation	Minimum	Maximum	Variables	HT Test		Conclusion
						Statistic	*p*-Value	
*CEE*	0.687	0.250	0.254	1.434	*CEE*	-	-	-
*ST*	49,015.270	81,336.180	228.000	709,725.000	ln*ST*	0.047	0.0000	stationary
*ED*	47,275.110	26,846.980	3005.920	164,220.000	ln*ED*	0.094	0.0000	stationary
*PC*	2844	1181	649	5967	ln*PC*	0.938	0.0014	stationary
*PU*	57.025	13.141	29.110	89.600	ln*PU*	−0.074	0.0000	stationary
*IS*	42.079	8.278	15.800	62.000	ln*IS*	0.065	0.0000	stationary
*EC*	14,427.640	8760.397	1135.330	41,845.000	ln*EC*	−0.247	0.0000	stationary
*FI*	2.066	1.945	0.107	12.099	ln*FI*	0.220	0.0000	stationary

**Table 3:** Marker values for different industrialization stages.

Basic Indicators	Pre-Industrialization	Industrialization Realization Stages			Post-Industrialization
		Early Industrialization	Mid-Industrialization	Late-Industrialization	
Economic development level (GDP per capita)					
1995 USD	610–1220	1220–2430	2430–4870	4870–9120	>9120
2020 USD	970–1930	1930–3850	3850–7700	7700–14,430	>14,430
Industrial structure					
Output value structure of three industries	*A* > *I*	*A* > 20%, *A* < *I*	*A* < 20%, *I* > *S*	*A* < 10%, *I* > *S*	*A* < 10%, *I* < *S*
Manufacturing structure					
Total manufacturing value added as a proportion of total merchandise value	<20%	20–40%	40–50%	50–60%	>60%
Spatial structure					
population urbanization rate	<30%	30–50%	50–60%	60–75%	>75%
employment structure					
Percentage of labors employed in primary sector	>60%	45–60%	30–45%	10–30%	<10%

Notes: The standard value of GDP per capita was calculated based on the GDP deflator. *A*, *I*, and *S* denote the proportion of the value-added of primary, secondary, and tertiary industries in GDP. Manufacturing value added/total merchandise production value added was mainly the ratio of industrial value added to value added by primary and secondary industries. Data processing and stage judgments were compared to three decimal places for data that were at critical values.

**Table 4:** Division of industrialization stages in 2020.

Industrialization Stages	Provinces
Mid-industrialization	Hainan, Heilongjiang, Guangxi, Guizhou, Yunnan, Gansu
Late-industrialization	Hebei, Inner Mongolia, Jilin, Hunan, Qinghai, Xinjiang, Shanxi, Liaoning, Jiangsu, Anhui, Fujian, Jiangxi, Shandong, Henan, Hubei, Guangdong, Chongqing, Sichuan, Shaanxi, Ningxia, Zhejiang
Post-industrialization	Beijing, Tianjin, Shanghai

**Table 5:** *CEE* input-output indicator system.

Indicators	First Grade Indexes	Second Grade Indexes	Unit
Input indicators	Capital input	Capital stocks	10^8^ RMB
	Labor input	Urban working population	10^4^ persons
	Energy input	Total energy consumption	10^4^ tons of standard coal
Output indicators	Desired output	GDP	10^8^ RMB
	Undesired output	CO_2_ emissions	10^4^ tons

**Table 6:** *CEE*’s regression results.

Variables	RE Model	FE Model	FE-tw Model
ln*ST*	0.0353 * (1.93)	0.1334 *** (13.00)	−0.0160 (−0.38)
ln*ED*	0.1215 *** (4.33)	0.2988 *** (7.27)	0.2518 *** (6.87)
ln*PC*	−0.2502 *** (−5.17)	−0.1008 *** (−3.82)	−0.2102 *** (−3.64)
ln*PU*	−0.0907 (−0.62)	−0.2343 ** (−2.64)	−0.1662 (−0.73)
ln*IS*	0.5002 *** (5.34)	−0.3765 *** (−6.89)	0.7124 *** (4.37)
ln*EC*	−0.0286 * (−1.83)	−0.0279 (−1.79)	−0.0234 * (−1.81)
ln*FI*	0.1062 *** (6.31)	0.0715 *** (6.46)	0.0404 * (2.20)
*cons*	1.4226 ** (2.33)	−2.5982 *** (−5.99)	1.3339 (1.03)
*R* ^2^	0.36	0.72	0.85
*F*-statistic	-	51.21	39.37

Note: “***”, “**”, “*” denote 1%, 5%, and 10% significance levels; values in brackets are *t*-statistics or *Z*-values, “-” are items not involved.

**Table 7:** Regression results of provincial *CEE* at different industrialization stages.

Variables	Mid-Industrialization			Late-Industrialization			Post-Industrialization		
	RE	FE	FE-tw	RE	FE	FE-tw	RE	FE	FE-tw
ln*ST*	−0.1341 *** (8.68)	−0.1464 *** (−4.47)	0.0369 (0.59)	0.0881 *** (3.86)	0.1480 *** (11.39)	−0.1070 ** (−2.01)	0.2624 ** (2.50)	0.2200 (0.97)	0.3011 (0.86)
ln*ED*	0.0447 (0.32)	0.1875 *** (2.72)	0.1455 ** (2.38)	0.1454 *** (4.12)	0.2957 *** (6.07)	0.2633 *** (6.10)	0.1646 ** (2.56)	0.6320 (2.53)	0.5161 (1.69)
ln*PC*	−0.4159 *** (11.06)	−0.3787 *** (−10.57)	−0.1626 ** (−2.02)	−0.2313 *** (−4.10)	−0.0574 (−1.44)	−0.1104 (−1.54)	−0.9846 *** (−4.44)	−0.5465 (−1.10)	−0.4156 (−0.75)
ln*PU*	0.9668 *** (8.68)	0.8022 *** (6.73)	0.7352 ** (2.53)	−0.4233 * (−1.88)	−0.0586 (−0.39)	1.2451 ** (3.03)	−3.7436 * (−1.78)	−5.2674 (−1.70)	−7.2689 * (−1.82)
ln*IS*	0.6306 *** (7.99)	0.5252 *** (3.69)	0.7480 *** (4.00)	1.1620 *** (6.52)	−0.3562 ** (−2.14)	1.1985 *** (4.43)	1.0236 *** (2.85)	0.4068 (0.65)	0.1132 (0.07)
ln*EC*	−0.0009 (−0.05)	0.0068 (0.35)	0.0062 (0.37)	−0.0311 * (−1.66)	−0.0293 (−1.49)	−0.0124 (−0.75)	−0.1888 * (−1.88)	−0.1498 (−0.86)	−0.0288 (−0.13)
ln*FI*	0.0582 *** (5.58)	0.0555 *** (4.19)	0.0002 (0.01)	0.0740 ** (3.46)	0.0704 *** (4.47)	0.0315 (1.41)	−0.0595 (−1.42)	−0.0045 (−0.09)	−0.0056 (−0.10)
*cons*	0.6510 (1.36)	−0.4331 (−0.50)	−3.1732 * (−1.94)	2.1685 ** (2.65)	−3.7042 *** (−4.45)	−4.8200 ** (−2.71)	20.7779 * (2.20)	18.3408 (1.35)	26.7429 (1.59)
*R* ^2^	0.82	0.89	0.93	0.53	0.72	0.84	0.69	0.83	0.84
*F*-statistic	-	24.65	31.10	-	33.85	31.67	-	5.01	4.30

Note: “***”, “**”, and “*” denote 1%, 5%, and 10% significance levels; values in brackets are *t*-statistics or *Z*-values, and “-” are unrelated items.

**Table 8:** Robustness test results.

Variables	*q*10	*q*25	*q*50	*q*75	*q*90
ln*ST*	0.1040 *** (3.02)	0.1064 *** (4.58)	0.0682 *** (4.83)	0.0739 *** (4.43)	0.0803 *** (5.76)
ln*ED*	0.0526 (0.91)	0.1726 *** (3.53)	0.1466 *** (2.87)	0.0978 (1.16)	0.0630 (0.90)
ln*PD*	−0.1659 ** (−2.55)	−0.2498 *** (−4.34)	−0.2072 *** (−3.33)	−0.2681 *** (−6.18)	−0.2286 *** (−4.59)
ln*PU*	−0.5655 *** (−3.50)	−0.4865 *** (−3.58)	0.0909 (0.68)	0.1475 (0.75)	0.1638 (0.73)
ln*IS*	0.2606 (1.45)	−0.1426 (−0.76)	−0.0403 (−0.59)	0.0580 (0.88)	0.0181 (0.26)
ln*EC*	−0.0089 (−0.31)	−0.0297 (−0.96)	−0.0213 (−1.14)	−0.0355 (−1.39)	−0.0635 ** (−2.31)
ln*FI*	0.1699 *** (4.43)	0.1332 *** (4.81)	0.1248 *** (7.37)	0.1058 *** (4.71)	0.1000 *** (4.23)
*cons*	2.2205 *** (2.68)	1.1603 (1.41)	−0.6624 (−1.18)	0.3323 (0.55)	0.5790 (1.01)

Note: “***” and “**” denote 1% and 5% significance levels; values in brackets are *t*-statistics or *Z*-values.

## Data Availability

The data used to support the findings of this study are available from the corresponding author upon request.
